# Chemical Characterization and Bioactive Potential of *Lippia alba* Essential Oil: Ethnobotanical Relevance, Antioxidant, Antifungal, Antibacterial, and Molecular Docking Studies

**DOI:** 10.3390/molecules31132284

**Published:** 2026-06-30

**Authors:** Juan E. Valdiviezo-Campos, Ramiro Fiestas-Jacinto, Karyn A. Olascuaga-Castillo, Segundo G. Ruiz-Reyes, Roger A. Rengifo-Penadillos, Junior F. Siguas-Peña

**Affiliations:** 1 Research in Natural Products, Programa Académico de Farmacia y Bioquímica, Facultad de Ciencias de la Salud, Universidad Privada Norbert Wiener, Lima 15046, Peru; jvaldiviezo@uwiener.edu.pe (J.E.V.-C.); ramiro.fiestas@uwiener.edu.pe (R.F.-J.); 2 Pharmascience Research Group, Pharmacology Laboratory, School of Human Medicine, Universidad Privada Antenor Orrego, Trujillo 13001, Peru; kolascuagac1@upao.edu.pe; 3 Department of Pharmacotechnics, Faculty of Pharmacy and Biochemistry, National University of Trujillo, Trujillo 13011, Peru; sruizr@unitru.edu.pe; 4 Department of Biochemistry, Faculty of Pharmacy and Biochemistry, National University of Trujillo, Trujillo 13011, Peru; rrengifo@unitru.edu.pe

**Keywords:** *Candida albicans*, *Staphylococcus aureus*, antioxidant activity, essential oil, in silico studies, bioactivity

## Abstract

*Lippia alba* (Mill.) N.E. Br. ex Britton & P. Wilson is an aromatic medicinal plant widely used in traditional medicine for the management of digestive, inflammatory, and infectious disorders. The present study aimed to investigate the ethnobotanical relevance, chemical composition, antioxidant, antifungal, antibacterial, and molecular docking properties of *L. alba* essential oil (EOLA). Ethnobotanical information was collected through semi-structured interviews with herbal vendors. The essential oil was obtained by hydrodistillation and chemically characterized by GC–MS and GC–FID, leading to the identification of 27 volatile constituents. The chemical profile was dominated by oxygenated monoterpenes (83.83%), with (−)-carvone (63.79%) and dihydrocarvyl acetate (17.74%) as the major compounds, confirming a carvone chemotype. EOLA exhibited moderate antioxidant activity, with values of 24.1 mg TE/100 g and 34.5 mg TE/100 g in the DPPH and ABTS assays, respectively. Furthermore, the essential oil demonstrated significant concentration-dependent antifungal and antibacterial activities against *Candida albicans* and *Staphylococcus aureus*. At the highest concentration tested, the antimicrobial activity approached that of the reference drugs. In silico ADMET analysis predicted favorable pharmacokinetic and drug-likeness properties for the major volatile constituents. Molecular docking studies revealed relevant interactions between several compounds, particularly α-gurjunene, alloaromadendrene, trans-α-bisabolene, and (−)-β-bourbonene, and molecular targets associated with oxidative stress and microbial inhibition, providing mechanistic insights into the biological activities observed experimentally. Overall, these findings support the ethnopharmacological use of *L. alba* and highlight its essential oil as a promising natural source of bioactive compounds with antioxidant and antimicrobial potential for future phytopharmaceutical applications.

## 1. Introduction

*Lippia alba* (Mill.) N.E. Br. ex Britton & P. Wilson, belonging to the Verbenaceae family, is an aromatic plant species widely distributed throughout Latin America and recognized for its richness in volatile secondary metabolites and its extensive use in traditional medicine [1,2]. In several countries, it has become one of the most commonly used medicinal plants under popular names such as “erva-cidreira”, “pronto alivio”, “*lippia*”, “santa maría”, and “juanilama” [3,4]. Numerous ethnobotanical studies have documented its use in the treatment of gastrointestinal, respiratory, nervous, and parasitic disorders, highlighting its therapeutic importance in rural and indigenous communities [5,6,7,8]. Moreover, ethnopharmacological investigations have demonstrated a strong correlation between its traditional uses and experimentally validated biological activities.

From a phytochemical perspective, the essential oil of *L. alba* exhibits remarkable chemical variability associated with the presence of multiple chemotypes, predominantly characterized by monoterpenes such as citral, linalool, carvone, limonene, and geraniol [9,10,11]. These volatile constituents have been extensively identified using advanced analytical techniques, particularly gas chromatography coupled with mass spectrometry (GC–MS) [9,11]. The chemical composition of *L. alba* essential oil is strongly influenced by several factors, including geographical origin, environmental conditions, phenological stage, and extraction methods, contributing to significant qualitative and quantitative variations among populations [9,12].

This chemical diversity has been associated with a broad spectrum of biological activities. In this regard, recent studies have demonstrated a remarkable antioxidant capacity of *L. alba* essential oil, which has been evaluated through the quantification of phenolic and flavonoid compounds [11]. These findings suggest its potential as a protective agent against oxidative stress in both plant systems and human cancer cells [3,4,13].

In addition to its antioxidant properties, *L. alba* has shown well-documented antimicrobial effects. The essential oil and its major constituents, particularly citral and carvone, have been reported to significantly inhibit the growth of pathogenic bacteria, including *Staphylococcus aureus* [14,15], as well as interfere with bacterial biofilm formation [14]. Likewise, considerable antifungal activity has been described against dermatophytes and phytopathogenic fungi, partially attributed to its linalool content [16,17,18]. These findings reinforce the potential application of *L. alba* as a natural alternative for the development of plant-derived antimicrobial agents.

Scientific interest in this species has also extended to computational and pharmacological studies, which suggest that the volatile metabolites of *L. alba* may interact with key enzymes of the nervous system, such as acetylcholinesterase, opening new therapeutic perspectives for neurodegenerative disorders [5,12]. Furthermore, recent investigations have explored its antiparasitic potential in aquaculture and veterinary systems, supporting the broad pharmacodynamic properties of these species across multiple biological models [7,19].

Although several studies have investigated the biological properties of aqueous, ethanolic, methanolic, and hydroalcoholic extracts of *Lippia alba*, the present study focused on its essential oil because this species is widely recognized as an aromatic medicinal plant whose pharmacological properties are largely attributed to volatile metabolites. Previous phytochemical investigations have identified monoterpenes and sesquiterpenes, particularly citral, carvone, linalool, and limonene, as major bioactive constituents associated with antimicrobial, antioxidant, anti-inflammatory, and neuropharmacological activities. As the essential oil represents the principal reservoir of these compounds, its characterization provides valuable insight into the chemical basis underlying the traditional medicinal uses of *L. alba*. Based on the ethnobotanical applications reported for this species, particularly those related to inflammatory conditions, digestive disorders, general wellness, and infection-related ailments, we hypothesized that its essential oil would exhibit antioxidant and antimicrobial activities associated with the presence of bioactive volatile constituents.

Despite the extensive literature on the phytochemistry and biological activities of *L. alba*, considerable intraspecific variability has been reported among geographical populations, resulting in distinct chemotypes and differences in biological activity. Consequently, the pharmacological properties described for one population cannot be directly extrapolated to others. Furthermore, carvone-rich chemotypes have been associated with biological activities that may differ from those reported for citral, linalool or limonene-dominated populations. Therefore, we further hypothesized that the biological properties of the *L. alba* population from northern Peru would be related to its specific chemical profile and major volatile constituents. In addition, studies integrating ethnobotanical information, chemical characterization, antioxidant and antimicrobial evaluations, and molecular docking analyses remain scarce for Peruvian populations. Therefore, the present study aimed to provide a comprehensive assessment of the ethnobotanical relevance, chemical composition, antioxidant and antimicrobial activities, and potential molecular mechanisms of *L. alba* essential oil from northern Peru, contributing to a better understanding of the biological significance of this regional chemotype.

## 2. Results and Discussion

**Figure 1:**
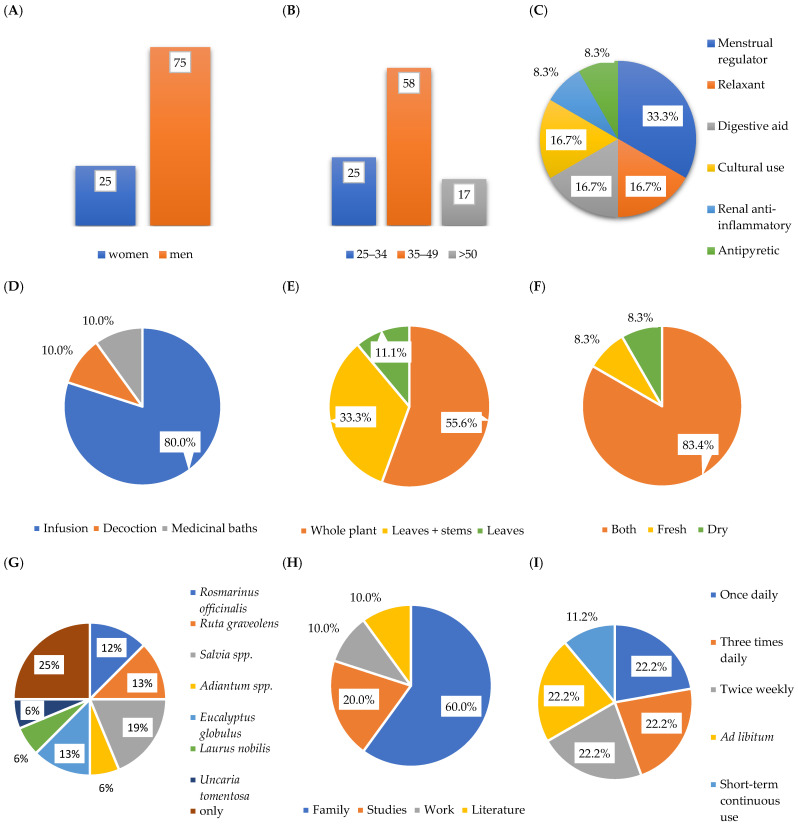
Ethnobotanical information recorded for *Lippia alba* from traditional herbal vendors. The panels illustrate the distribution of respondents according to (**A**) sex; (**B**) age group; (**C**) traditional medicinal applications; (**D**) mode of administration; (**E**) plant parts utilized; (**F**) health conditions treated; (**G**) use in combination with other medicinal plants; (**H**) origin of traditional knowledge; and (**I**) dosage practices.

The demographic profile of the informants revealed a predominance of women (75%) and individuals aged 35–49 years (58%) (Figure 1A,B). These findings highlight the central role of adult women in the preservation and transmission of ethnobotanical knowledge related to *Lippia alba*. Similar patterns have been reported for other species of the genus *Lippia*, where women are recognized as primary custodians of medicinal plant knowledge and household healthcare practices. For instance, Oliveira et al. [20] reported that *Lippia origanoides* is predominantly used by women in Brazilian communities for the treatment of genitourinary and digestive disorders. Likewise, previous studies indicate that traditional knowledge regarding the sedative, digestive, and antimicrobial uses of *L. alba* is mainly transmitted through female family members [2]. This predominance reflects the broader role of women in family care, herbal preparation, and subsistence practices, a pattern widely documented in ethnobotanical research [21]. Furthermore, the predominance of economically active adults suggests that traditional knowledge of *L. alba* remains dynamic and continues to be transmitted across generations [22].

The medicinal uses reported by informants were grouped into functional ethnobotanical categories. The most frequently cited use was related to gynecological applications, particularly menstrual regulation (33.3%), followed by relaxant (16.7%) and digestive uses (16.7%). A considerable proportion of citations also corresponded to culturally defined conditions such as “mal aire” and “susto” (16.7%), whereas renal anti-inflammatory and antipyretic uses were reported less frequently (8.3% each) (Figure 1C). These findings are consistent with previous ethnopharmacological reports describing *L. alba* as a sedative, digestive, antispasmodic, and remedy for menstrual disorders [2]. Similar uses have been documented for other *Lippia* species, including *L. origanoides* and *L. graveolens*, which are traditionally employed for gastrointestinal complaints, inflammatory conditions, and culturally defined illnesses [20,23]. The prominence of gynecological and relaxant applications reinforces the traditional role of *L. alba* in women’s health and nervous system regulation, which may be associated with bioactive compounds such as citral and linalool, previously linked to antispasmodic and anxiolytic effects. More broadly, *L. alba* has been reported in traditional medicine for the management of diarrhea, fever, inflammation, respiratory disorders, and body pain, highlighting its wide therapeutic versatility [24,25].

Regarding preparation methods, infusion was by far the most frequently reported form of administration (80%), followed by decoction (10%) and medicinal baths (10%) (Figure 1D). The predominance of infusion is consistent with the use of aromatic species rich in volatile compounds and agrees with previous studies on *L. alba* and other medicinal plants, where herbal teas prepared by infusion or decoction constitute the most common form of use [6]. Ethnobotanical surveys conducted throughout Latin America have similarly identified infusion as the dominant preparation method, accounting for up to 73.4% of reported uses [26]. In contrast, studies from North Africa have reported a higher prevalence of decoction, suggesting that preparation practices may vary according to cultural traditions and plant characteristics [27]. Overall, the predominance of infusion observed in the present study reflects a global tendency to employ simple, accessible, and effective extraction methods for medicinal plants rich in volatile and thermolabile constituents [28].

The whole plant was the most frequently utilized plant part (55.6%), followed by the combined use of leaves and stems (33.3%), whereas the exclusive use of leaves was less common (11.1%) (Figure 1E). The preference for using the entire plant may reflect a traditional strategy aimed at maximizing the extraction of bioactive compounds. Similar practices have been reported for *L. alba*, whose aerial parts are commonly used to treat digestive and nervous disorders [2]. However, studies on other *Lippia* species indicate a more selective use of leaves and flowering tops, tissues generally characterized by higher concentrations of essential oils and secondary metabolites [20,23,25,29].

The condition of use was predominantly reported as either fresh or dried material (83.4%), whereas exclusive use of fresh or dried plants was mentioned by only 8.3% of informants each (Figure 1F). This flexibility is consistent with previous ethnobotanical reports and reflects the adaptability of traditional practices to plant availability [30]. Phytochemical studies have demonstrated that drying may alter the relative abundance of volatile constituents without necessarily compromising biological activity. For example, investigations on *Lippia thymoides* and *Lippia origanoides* showed that drying can affect essential oil yield and chemical composition, while antioxidant activity is generally preserved under appropriate processing conditions [31,32]. Similar observations have been reported for *L. alba*, whose biological properties are largely associated with relatively stable compounds such as citral, linalool, and carvone [3,33].

The use of *Lippia alba* in combination with other medicinal plants was reported by 75% of informants, whereas only 25% indicated its use as a single species (Figure 1G). The species most frequently associated with *L. alba* included *Rosmarinus officinalis*, *Ruta graveolens*, *Salvia* spp., and *Adiantum* spp., all of which are traditionally recognized for their aromatic, anti-inflammatory, and ritual applications. The frequent use of plant mixtures suggests a synergistic approach in traditional medicine, particularly for medicinal baths and the treatment of culturally defined conditions. Similar practices have been widely documented in ethnobotanical studies, where combinations of medicinal plants are employed to enhance therapeutic efficacy and address multifactorial health conditions [34,35]. Recent evidence further suggests that interactions among secondary metabolites may produce additive or synergistic effects, particularly in formulations rich in essential oils and terpenoids [36,37]. Comparable patterns have been reported for other species of the genus *Lippia*, where aromatic plant mixtures are traditionally used for digestive, inflammatory, and nervous disorders [8,38].

Family-based transmission was identified as the principal source of ethnomedicinal knowledge regarding *L. alba* (60%), followed by formal education (20%), books and internet resources (10%), and work-related experience (10%) (Figure 1H). These findings are consistent with previous ethnobotanical studies showing that traditional medicinal knowledge is predominantly transmitted orally through family networks, although it is increasingly complemented by formal and scientific information sources [34,39]. This coexistence of traditional and scientific knowledge reflects the development of hybrid knowledge systems that contribute to the preservation and adaptation of ethnomedicinal practices. Nevertheless, such knowledge is often concentrated among older generations and may be progressively lost due to declining interest among younger populations. As reported by Megersa et al. [29], ethnomedicinal knowledge is primarily acquired through family members and close social networks, but its intergenerational transmission is currently facing important challenges.

Considerable variability was observed in dosage and treatment duration among informants. The most frequently reported administration patterns included once daily (22.2%), three times daily (22.2%), and twice-weekly use (22.2%), whereas an additional 22.2% reported ad libitum consumption as “agua de tiempo” (Figure 1I). Treatment duration generally ranged from 3 to 10 days, although half of the respondents did not specify a defined period of use. Such variability is characteristic of traditional medicinal systems, where dosage is rarely standardized and is typically based on empirical knowledge, availability of plant material, preparation methods, and individual patient characteristics [40]. In many traditional contexts, dosage is expressed using informal measurements such as cups, spoons, or handfuls and is adjusted according to age, sex, and perceived health status.

The absence of standardized dosage regimens has been identified as an important limitation of traditional medicine because it may contribute to inconsistent therapeutic outcomes and potential risks associated with under- or over-administration [41,42]. Nevertheless, traditional healers often employ corrective practices, including the combination of different plant materials, to improve efficacy and reduce adverse effects [43]. Furthermore, the well-documented phytochemical variability of *L. alba*, including differences in chemotype and metabolite composition, may influence perceived efficacy and contribute to the diversity of dosage practices observed among communities. These findings highlight the adaptive and context-dependent nature of dosage determination in traditional medicinal systems [8].

Overall, the ethnobotanical information obtained in this study provides an important framework for interpreting the biological activities of *L. alba*. Traditional uses reported by herbal vendors, particularly those related to infectious, inflammatory, digestive, and general wellness conditions, are consistent with the antioxidant and antimicrobial activities demonstrated by the essential oil. Although ethnobotanical evidence alone cannot establish pharmacological efficacy, the convergence between traditional knowledge and experimental findings reinforces the ethnopharmacological relevance of this species and supports its continued investigation as a source of bioactive natural products.

The essential oil of *Lippia alba* exhibited antioxidant activities of 24.10 ± 0.10 mg TE/g and 34.53 ± 0.11 mg TE/g as determined by the DPPH and ABTS assays, respectively (Table 1). These values indicate a moderate radical-scavenging capacity, with a higher response in the ABTS assay, suggesting a broader reactivity toward both hydrophilic and lipophilic radical species. This behavior is commonly reported for essential oils due to the diverse polarity and reactivity of their volatile constituents. The antioxidant activity observed in the present study is consistent with previous reports for *L. alba* and other species of the Verbenaceae family, in which substantial variability has been associated with differences in chemotype, environmental conditions, extraction procedures, and analytical methodologies.

In general, essential oils exhibit lower antioxidant capacity than polar plant extracts because they contain relatively low concentrations of phenolic compounds, which are among the most effective natural antioxidants. For example, methanolic extracts of *Lippia schomburgkiana* have shown antioxidant values as high as 327.0 ± 24.8 mg TE/g, demonstrating that the polar fraction concentrates most of the antioxidant constituents [44]. Likewise, several studies have reported significantly greater antioxidant activity in phenolic-rich ethanolic or methanolic extracts of *Lippia* species compared with their corresponding essential oils.

Studies specifically focused on essential oils have generally reported lower antioxidant values. The antioxidant capacity of *L. alba* essential oil has been estimated at approximately 51.9 mg TE/g, although this value varies according to chemotype and extraction conditions [45]. Similarly, antioxidant activities ranging from 131.1 to 336.0 mg TE/g have been described for *Lippia grandis* in TEAC/ABTS systems [46]; however, these results were obtained under different experimental conditions and frequently involved enriched extracts, limiting direct comparisons. Furthermore, Reyes-Solano et al. [47] reported IC_50_ values of 12.45 ± 0.57 mg/mL (DPPH) and 15.6 ± 0.1 µmol/g (ABTS) for hydrodistilled *L. alba* essential oil, confirming the moderate antioxidant performance typically associated with this matrix. In contrast, studies evaluating leaf extracts and different *L. alba* accessions have reported substantially stronger radical-scavenging activity, with IC_50_ values in the low µg/mL to low mg/mL range depending on solvent polarity and genotype [48]. Recent investigations have further demonstrated that the pronounced chemical variability among *Lippia* genotypes significantly influences their antioxidant potential [49].

The antioxidant activity observed in the present study falls within the expected range for essential oils and can be largely attributed to the presence of oxygenated monoterpenes, particularly carvone, together with minor constituents such as linalool. Oxygenated monoterpenes are known to contribute to antioxidant activity through mechanisms involving hydrogen atom donation, electron transfer, and stabilization of reactive oxygen species [50,51]. Nevertheless, their antioxidant effectiveness is generally lower than that of phenolic monoterpenes such as thymol or carvacrol, which possess stronger reducing and radical-scavenging properties. Consequently, the moderate antioxidant activity observed for the analyzed oil is consistent with its carvone-dominated chemotype.

Methodological factors must also be considered when comparing antioxidant data across studies. Parameters such as solvent composition, radical concentration, incubation time, and reaction kinetics can significantly affect the measured response [52,53]. Moreover, the DPPH and ABTS assays differ in their sensitivity toward compounds of varying polarity, which may explain the higher antioxidant values obtained with the ABTS method. Therefore, comparisons among studies should be interpreted cautiously, taking into account both chemical composition and experimental design.

Although DPPH and ABTS are widely accepted methods for assessing radical-scavenging activity, they evaluate only a limited aspect of antioxidant behavior. Additional assays based on electron-transfer mechanisms, such as FRAP and CUPRAC, would provide complementary information regarding the reducing capacity of *L. alba* essential oil and contribute to a more comprehensive evaluation of its antioxidant potential.

Overall, the results confirm that *L. alba* essential oil possesses the ability to neutralize free radicals and reduce oxidant species; however, its antioxidant activity should be regarded as a secondary biological property rather than its principal pharmacological attribute. This finding is expected considering the predominance of carvone and other oxygenated monoterpenes in the oil, together with the absence of highly reducing phenolic constituents. Nevertheless, this moderate antioxidant capacity may still be relevant in phytopharmaceutical applications, where it could contribute to the oxidative stability of formulations and act synergistically with the well-documented antimicrobial properties of the essential oil.

Therefore, the antioxidant potential of EOLA should be interpreted as a complementary biological property rather than its principal pharmacological attribute. While its practical relevance as a standalone antioxidant agent may be lower compared to other plant-derived alternatives rich in phenolic compounds, its radical-scavenging capacity provides significant added value. In potential phytopharmaceutical applications, this moderate antioxidant activity could help prevent the auto-oxidation of formulations and act synergistically to support the more potent antimicrobial properties of the essential oil.

**Figure 2:**
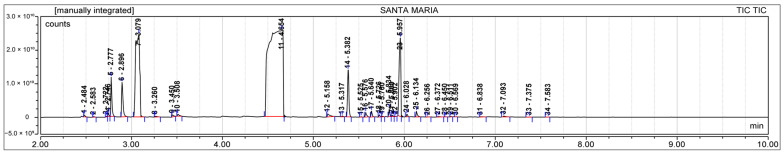
GC–MS chromatographic profile of volatile constituents detected in *Lippia alba* essential oil (EOLA).

The chemical composition of the essential oil obtained from *Lippia alba* was characterized by GC–MS analysis, leading to the identification of 27 compounds that accounted for the majority of the volatile fraction (Figure 2). The predominant constituent was (−)-carvone (63.79%), followed by dihydrocarvyl acetate (17.74%), α-cubebene (3.40%), α-gurjunene (2.94%), and β-pinene (2.12%). Minor constituents included linalool (1.92%), terpinolene (1.16%), and several sesquiterpenes present in low proportions. In terms of chemical classes, the essential oil was dominated by oxygenated monoterpenes (83.83%), followed by monoterpene hydrocarbons (5.93%) and sesquiterpenes (7.90%) (Table 2). This profile indicates a clear predominance of oxygenated compounds, mainly associated with carvone and its related derivatives.

Based on its chemical composition, the analyzed sample can be classified as a carvone chemotype, one of the most frequently reported chemotypes of *L. alba*. Previous studies have demonstrated remarkable chemical diversity within this species, with carvone-, citral-, linalool-, and limonene-rich chemotypes representing the most characteristic profiles [12,49,54,55]. The composition observed in the present study differs considerably from citral-rich chemotypes reported in Brazil and Colombia, where geranial and neral frequently constitute more than 60–80% of the essential oil, and from linalool-rich chemotypes characterized by high concentrations of linalool and related oxygenated monoterpenes [54,55]. Likewise, limonene-carvone chemotypes described in several South American populations generally exhibit a more balanced distribution between limonene and carvone, whereas the present sample showed a marked predominance of carvone accompanied by substantial amounts of dihydrocarvyl acetate [56].

The elevated proportion of carvone recorded in this study is consistent with previous reports indicating that this monoterpene may account for more than 50% of the essential oil composition in carvone-type populations of *L. alba* [56,57]. Such predominance suggests a marked biosynthetic orientation toward oxygenated cyclic monoterpenes. However, although the carvone chemotype is generally regarded as relatively stable, its composition may be influenced by environmental, geographical, agronomic, and genetic factors [58]. Malik et al. [58] emphasized that variations in ecological conditions and cultivation practices can significantly affect the accumulation of volatile metabolites in *L. alba*. Similarly, Ricciardi et al. [59] reported pronounced differences in the relative abundance of major metabolites across Latin American populations, highlighting chemotypic diversity as one of the defining characteristics of this species. Consequently, the biological properties reported for one population cannot be directly extrapolated to another without prior phytochemical characterization.

The expression of a particular chemotype is influenced by multiple intrinsic and extrinsic factors. Previous studies have demonstrated that geographical origin, altitude, climatic conditions, temperature, rainfall, solar radiation, soil characteristics, seasonality, and genetic variability can significantly affect both essential oil yield and chemical composition [58,60,61]. Seasonal fluctuations may alter the relative abundance of monoterpenes and sesquiterpenes, while environmental stress conditions can modify secondary metabolite biosynthesis. In addition, the plant organ used for extraction is another important source of variability. Leaves generally contain higher concentrations of monoterpenes, whereas stems, flowers, and reproductive structures may exhibit distinct metabolite profiles [12]. Post-harvest processing and extraction methods may also influence the relative abundance of volatile constituents, contributing to the compositional differences reported among studies [60]. Therefore, the chemical profile observed in the present investigation likely reflects the combined influence of the environmental conditions of northern Peru and the genetic characteristics of this regional population.

From a biological perspective, the predominance of oxygenated monoterpenes is particularly relevant because these metabolites are strongly associated with the pharmacological properties of *L. alba* essential oils. Previous investigations have demonstrated that biological activity is largely chemotype-dependent, with carvone- and citral-rich chemotypes exhibiting pronounced antimicrobial and antifungal activities, whereas linalool-rich chemotypes are more frequently associated with sedative and anxiolytic effects [11,61]. Accordingly, the high concentration of carvone and related oxygenated monoterpenes identified in the present study likely contributes significantly to the antioxidant and antimicrobial activities observed for the analyzed oil.

In addition to the major constituents, minor metabolites such as linalool (1.92%) and terpinolene (1.16%) may also contribute to the biological properties of the essential oil. Although present in relatively low concentrations, these compounds have been reported to modulate antioxidant and antimicrobial activities through synergistic interactions with major constituents [12]. Likewise, the sesquiterpene fraction, represented by compounds such as α-cubebene and α-gurjunene, may contribute to the overall biological activity through additive or synergistic effects despite their lower abundance [11].

Overall, the results confirm that the analyzed essential oil corresponds to a highly concentrated carvone chemotype characterized by a predominance of oxygenated monoterpenes and comparatively low proportions of sesquiterpenes and hydrocarbon compounds. This phytochemical profile is consistent with previous reports for carvone-rich populations of *L. alba* and further supports the biological relevance of this chemotype, which has been repeatedly associated with antimicrobial, antioxidant, and neuropharmacological properties [11,61]. The focus on the essential oil was justified by the aromatic nature of *L. alba* and the recognized contribution of its volatile constituents to the biological activities traditionally attributed to the species. Since the major metabolites identified in this study correspond to volatile monoterpenes and sesquiterpenes, essential oil analysis represents an appropriate approach for evaluating the phytochemical profile and associated biological activities of this carvone-rich chemotype.

**Figure 3:**
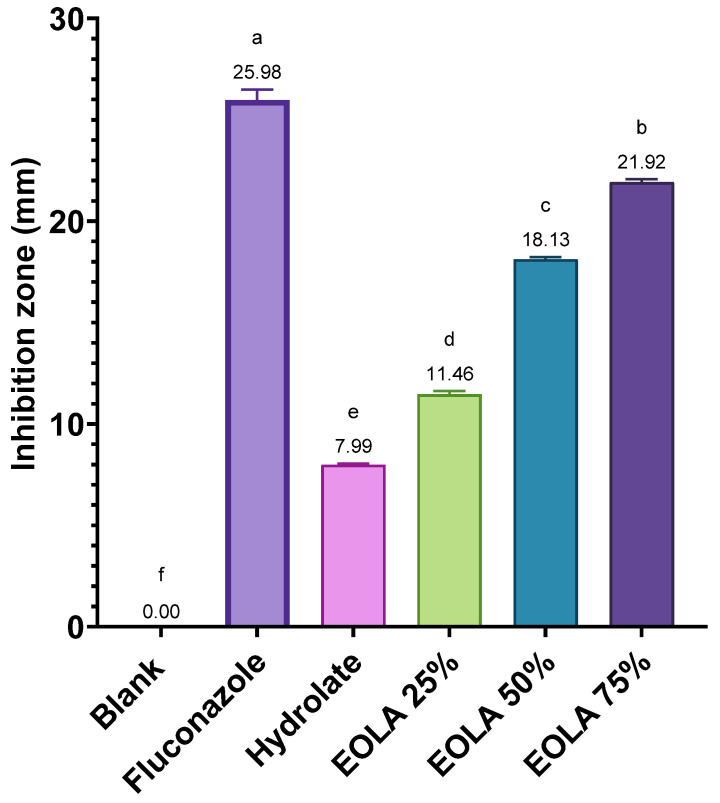
Antifungal activity of essential oil from the species *L. alba*; Means with different superscript letters are significantly different (*p* < 0.05); EOLA = *Lippia alba* essential oil.

The antifungal activity of *Lippia alba* essential oil (EOLA) against *Candida albicans* demonstrated a clear concentration-dependent inhibitory effect, confirming the biological relevance of its volatile constituents (Figure 3). The inhibition zones increased progressively from 11.46 mm at 25% EO to 21.92 mm at 75% EO, while fluconazole, used as the positive control, exhibited the highest inhibition zone (25.98 mm). In contrast, the negative control showed no inhibitory activity, confirming the reliability of the assay conditions. Notably, the antifungal effect observed at the highest EO concentration approached that of fluconazole, highlighting the considerable antifungal potential of the essential oil.

The concentration-dependent increase in fungal growth inhibition indicates that the antifungal activity is directly associated with the abundance of volatile bioactive metabolites present in the oil. These findings are consistent with previous studies reporting significant antifungal activity of *L. alba* essential oils, particularly citral- and carvone-rich chemotypes, against *Candida* spp. and other pathogenic fungi [14,61,62]. Mesa-Arango et al. [61] demonstrated that chemotypes rich in oxygenated monoterpenes exhibited pronounced antifungal activity against clinically relevant fungi, attributing this effect to membrane destabilization and disruption of fungal integrity. Interestingly, citral-rich chemotypes are often reported to exhibit stronger antifungal activity because geranial and neral readily interact with fungal membranes and ergosterol-associated pathways [61]. Nevertheless, the carvone-rich chemotype identified in the present study exhibited substantial antifungal activity, with inhibition zones reaching 21.92 mm at 75% EO, suggesting that carvone and related oxygenated terpenes can also contribute significantly to fungal growth inhibition.

The antifungal activity observed may be associated with the predominance of oxygenated monoterpenes identified in the GC–MS profile, particularly (−)-carvone, linalool, terpinolene, and β-pinene, together with minor sesquiterpenes. These metabolites are known to alter membrane permeability, disrupt ergosterol organization, and promote leakage of intracellular constituents, ultimately compromising fungal viability [50,63]. Previous studies have also demonstrated that *L. alba* essential oil can inhibit biofilm formation and interfere with the yeast-to-hyphae transition in *Candida*, reinforcing its antifungal relevance [18].

Comparable results were reported by Sabaly et al. [64], who evaluated the antifungal activity of *L. alba* essential oil against *Aspergillus flavus* and observed complete fungal inhibition at concentrations between 100 and 1000 ppm, mainly attributed to the predominance of geranial and neral. Similarly, Santos et al. [65] described significant antifungal activity for *L. alba* essential oil and its isolated constituents, highlighting the contribution of oxygenated terpenes to fungal membrane destabilization and growth inhibition. Together, these reports support the concentration-dependent antifungal effect observed in the present study. However, while previous findings on Aspergillus flavus highlight the isolated contribution of specific oxygenated terpenes, the bioactivity of EOLA against Candida albicans cannot yet be conclusively attributed to a single chemical class. It is highly probable that the observed antifungal efficacy results from a complex synergistic interaction among the dominant oxygenated monoterpenes, such as (−)-carvone, and minor sesquiterpene constituents, rather than the isolated action of a single compound. Further experimental studies involving the fractionation of the essential oil are required to definitively elucidate the active principles and their specific pharmacodynamic contributions.

Interestingly, molecular docking analyses supported the antifungal potential of EOLA by predicting favorable interactions between its volatile constituents and the selected fungal target. These findings suggest that the observed antifungal activity may result from the combined contribution of multiple compounds present in the essential oil rather than being exclusively associated with its major constituent. Such observations are consistent with the widely accepted concept that the biological activity of essential oils often arises from synergistic or additive interactions among their components. Similar behavior has been reported for different *Lippia alba* chemotypes, where both major and minor constituents contribute to the overall antifungal effectiveness of the oil [11,12].

The markedly lower antifungal activity observed for the hydrolate (7.99 mm) compared with the essential oil further supports the importance of the volatile lipophilic fraction as the principal source of antifungal compounds. Similar findings have been reported for hydrolates and aromatic waters, which generally exhibit weaker antimicrobial activity due to the reduced concentration of volatile terpenoids [66]. Furthermore, the present results agree with quantitative studies reporting low MIC and MFC values for *L. alba* essential oils against *Candida* species [18,62], suggesting that the inhibition zones observed here reflect biologically relevant antifungal activity rather than merely the diffusion properties of volatile compounds.

**Figure 4:**
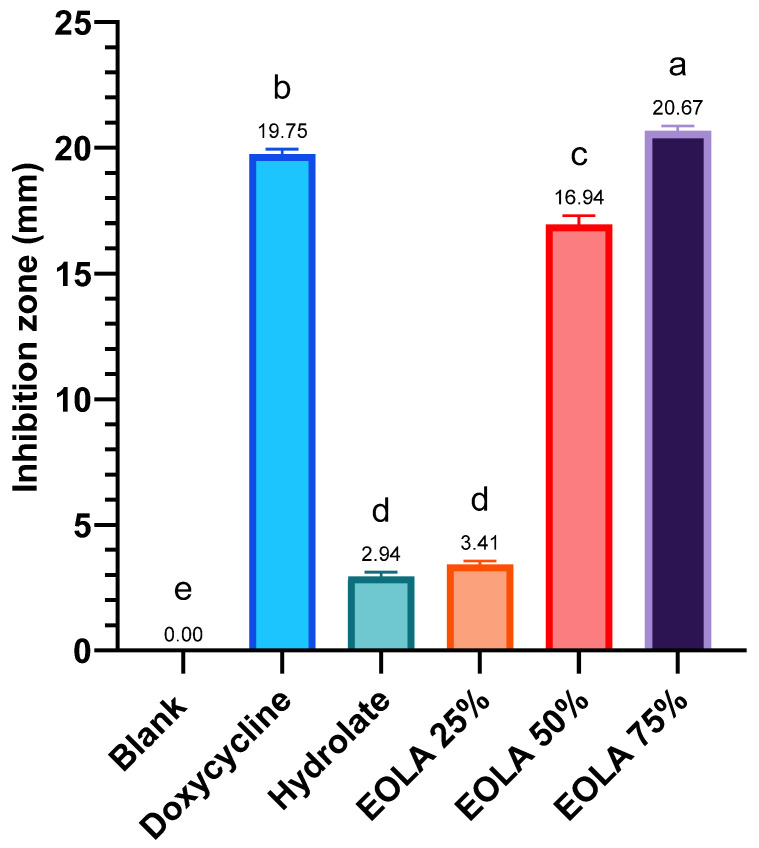
Antibacterial activity of essential oil from the species *L. alba*. Means with different superscript letters are significantly different (*p* < 0.05); EOLA = *Lippia alba* essential oil.

The antibacterial activity of *Lippia alba* essential oil (EOLA) against *Staphylococcus aureus* demonstrated a clear concentration-dependent inhibitory effect (Figure 4), confirming the biological relevance of its volatile constituents. Doxycycline, used as the positive control, exhibited an inhibition zone of 19.75 mm, validating both the susceptibility of the bacterial strain and the reliability of the assay conditions. In contrast, the negative control showed no inhibitory activity. EOLA produced inhibition zones of 3.41 mm, 16.94 mm, and 20.67 mm at 25%, 50%, and 75%, respectively. Notably, the highest EO concentration surpassed the inhibitory effect of doxycycline, indicating remarkable antibacterial potency against *S. aureus*. Conversely, the hydrolate exhibited considerably lower inhibition (2.94 mm), suggesting that the antibacterial activity is mainly associated with the lipophilic volatile fraction of the essential oil.

The concentration-dependent increase in bacterial growth inhibition indicates that the antibacterial activity is directly related to the abundance of volatile bioactive metabolites present in the EO. These findings are consistent with previous studies describing the strong antimicrobial potential of *L. alba*, particularly citral-, limonene-, and carvone-rich chemotypes, against clinically relevant bacterial strains [1,2]. Hennebelle et al. [2] reported that the antimicrobial activity of *L. alba* is mainly associated with oxygenated monoterpenes capable of disrupting bacterial membrane integrity and altering essential cellular metabolism. Similarly, Porfírio et al. [14] demonstrated that *L. alba* EO, as well as its major constituents citral and carvone, exhibited significant antibacterial and antibiofilm activity against *S. aureus*, with MIC and MBC values of 0.5 mg/mL and complete inhibition of biofilm formation at the same concentration.

The strong antibacterial activity observed in the present study appears to be consistent with the carvone-rich chemotype identified by GC–MS analysis. Although citral-rich chemotypes are frequently reported as the most potent antibacterial variants of *L. alba* due to the high reactivity of geranial and neral toward bacterial membranes, carvone-rich oils have also demonstrated relevant activity against Gram-positive bacteria, particularly *S. aureus* [14,67]. The inhibition zone of 20.67 mm obtained at 75% EO, which slightly exceeded that produced by doxycycline, suggests that the high abundance of (−)-carvone (63.79%), together with other oxygenated monoterpenes, contributes substantially to the antibacterial effect observed.

The elevated antibacterial activity may be associated with the predominance of oxygenated monoterpenes and terpene hydrocarbons identified in the GC–MS profile, including carvone, linalool, terpinolene, and β-pinene. Previous studies have demonstrated that citral exhibits antibacterial activity against methicillin-resistant *Staphylococcus aureus* strains, with MIC values ranging from 5 to 40 mg/mL and MBC values between 10 and 40 mg/mL [68]. Likewise, oxygenated monoterpenes are known to interact with bacterial phospholipid bilayers, increase membrane permeability, induce leakage of intracellular constituents, and impair essential metabolic processes such as ATP synthesis and bacterial respiration [69]. These mechanisms may explain the concentration-dependent antibacterial effect observed in the present study.

The antibacterial relevance of *L. alba* EO is further supported by Santos et al. [65], who identified antimicrobial volatile constituents in the essential oil and associated their activity with oxygenated monoterpenes capable of destabilizing bacterial membranes. Likewise, Batista et al. [70] reported that a geranial/neral-rich *L. alba* EO exhibited antibacterial and antibiofilm activity against pathogenic bacteria, including a rapid reduction in cell viability and MIC values around 1 mg/mL. Although their study focused on *Salmonella typhi* and *Shigella dysenteriae*, the proposed mechanism involving membrane disruption and metabolic impairment is consistent with the antibacterial activity observed in the present study.

Comparable antibacterial effects have also been reported by Nonato et al. [67], who demonstrated pronounced antibacterial activity of *L. alba* essential oils against multidrug-resistant *Staphylococcus aureus*, particularly in citral-rich chemotypes. The authors attributed this activity to the synergistic action of oxygenated monoterpenes capable of destabilizing bacterial membranes and interfering with essential metabolic pathways. Although the oil evaluated in the present study belongs to the carvone chemotype, the antibacterial potency observed was comparable to that reported for other bioactive chemotypes of *L. alba*, reinforcing the relevance of carvone-rich populations as potential sources of antimicrobial agents.

Molecular docking analyses provided additional support for the antibacterial activity of EOLA by indicating favorable interactions between its constituents and the selected bacterial target. These results suggest that the observed antibacterial effect may arise from the combined contribution of multiple volatile compounds, reinforcing the concept that the biological activity of essential oils is often associated with synergistic interactions among their constituents [11,12].

Overall, the present findings demonstrate that the antibacterial activity of EOLA is consistent with the biological profile expected for a carvone-rich chemotype, combining a high abundance of oxygenated monoterpenes with the potential contribution of minor sesquiterpenes. These results reinforce the potential application of *L. alba* essential oil in phytopharmaceutical, topical, and antimicrobial formulations targeting *Staphylococcus aureus*, including resistant strains.

In silico analyses performed using the SwissADME and pkCSM platforms were employed to assess the physicochemical and pharmacokinetic properties of the volatile constituents identified in EOLA.

A total of 27 compounds detected by GC–MS were included in the evaluation. The calculated molecular descriptors indicated that all analyzed metabolites complied with Lipinski’s Rule of Five, one of the most widely used criteria for predicting oral bioavailability and drug-likeness. As shown in Table 3, the compounds exhibited molecular weights below 500 g/mol, molar refractivity values within the range of 40–130, LogP values lower than 5, no more than five hydrogen-bond donors (HBD), and no more than ten hydrogen-bond acceptors (HBA). Collectively, these physicochemical characteristics suggest favorable membrane permeability and potential for intestinal absorption, supporting the drug-like profile of the major volatile constituents of *Lippia alba* essential oil.

These results are consistent with recent studies evaluating natural compounds through in silico ADME approaches, which have demonstrated that a significant proportion of plant-derived metabolites comply with Lipinski’s Rule of Five and exhibit favorable permeability, solubility, and pharmacokinetic profiles [71,72]. Recent large-scale ADMET analyses of phytochemicals have shown that compounds meeting Lipinski’s criteria tend to present adequate gastrointestinal absorption and low toxicity risk, supporting their potential as drug−like molecules.

Furthermore, recent reviews emphasize that in silico ADME screening has become a fundamental step in natural product−based drug discovery, as it allows early identification of compounds with suitable pharmacokinetic properties while reducing experimental costs and failure rates [73]. These approaches are especially relevant for volatile compounds such as terpenoids, whose physicochemical properties often align with drug-likeness parameters but may vary depending on structural complexity.

The ADME and toxicity predictions revealed generally favorable pharmacokinetic characteristics for the 27 volatile compounds identified in EOLA. All molecules showed high predicted human intestinal absorption (HIA), exceeding 93%, indicating a strong potential for oral absorption. Likewise, most compounds exhibited favorable blood–brain barrier (BBB) permeability, with predicted logBB values ranging from 0.5 to 0.9 and CNS permeability (logPS) values between −1 and −3, suggesting their ability to reach the central nervous system.

Regarding interactions with cytochrome P450 enzymes, the majority of compounds were not predicted to inhibit the evaluated isoforms. However, a subset of metabolites showed potential inhibitory activity toward CYP1A2, CYP2C19, and CYP2C9, while several compounds were also identified as possible CYP substrates. Toxicological predictions were generally favorable, as all compounds were classified as non-hepatotoxic and non-mutagenic according to the AMES model. Nevertheless, skin sensitization potential was predicted for some constituents, indicating the need for further toxicological evaluation before potential pharmaceutical or cosmetic applications. Detailed ADME and toxicity parameters are presented in Table 4.

**Figure 5:**
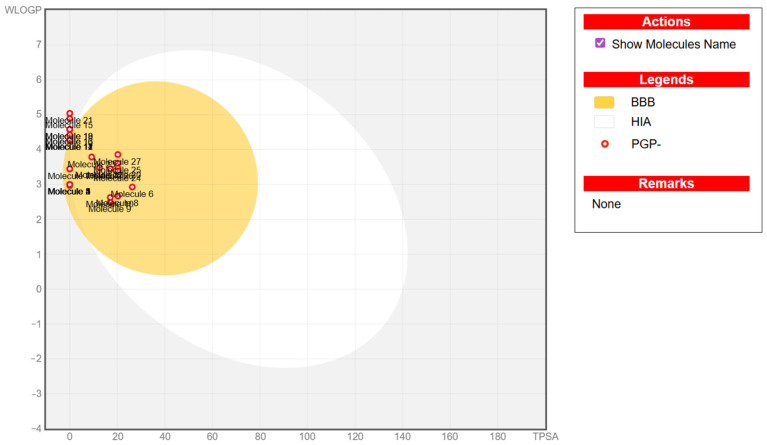
The BOILED-Egg model of 27 chemical compounds of EOLA.

The BOILED-Egg model (Figure 5), which integrates lipophilicity (WLOGP) and topological polar surface area (TPSA) to predict passive gastrointestinal absorption and brain penetration, further supported these findings. Most compounds were located within the regions associated with favorable absorption and distribution, whereas only a few metabolites were positioned at the margins or outside the optimal zones, suggesting reduced permeability. Overall, the model indicates that the majority of EOLA constituents possess physicochemical characteristics compatible with efficient intestinal absorption and passive penetration of the blood–brain barrier, highlighting their potential relevance for applications involving central nervous system targets.

Molecular docking was employed as a complementary computational approach to investigate potential molecular mechanisms underlying the biological activities observed experimentally. Because the antioxidant, antifungal, and antibacterial properties of *Lippia alba* essential oil (EOLA) had already been demonstrated through in vitro assays, the docking analysis was not intended to predict bioactivity per se, but rather to explore plausible interactions between the volatile constituents of the oil and biologically relevant molecular targets. Consequently, the calculated binding affinities should be interpreted as indicators of potential target engagement that may help explain, at the molecular level, the biological effects observed experimentally.

The docking study assessed the interactions of the 27 volatile constituents identified in EOLA with three protein targets related to antioxidant (2CDU), antifungal (4DKI), and antibacterial (5TZ1) activities. Several compounds displayed favorable binding affinities, with some docking scores approaching or exceeding those obtained for the reference ligands, including ascorbic acid (−6.2 kcal/mol), fluconazole (−6.9 kcal/mol), and oxacillin (−8.6 kcal/mol). The reference ligands displayed interaction patterns consistent with their known binding modes, supporting the suitability of the selected receptors for the proposed analyses. Specifically, ascorbic acid formed hydrogen bonds with His10, Thr112, Gly114, Ser115, Lys134, and Asp282; fluconazole established hydrogen bonds with Lys143 and Cys470, together with a π–sulfur interaction involving Cys470; whereas oxacillin interacted through hydrogen bonds with Tyr446, Thr600, and Gln521, as well as a π–sulfur interaction with Tyr446. These interaction profiles are indicative of stable ligand–protein complexes, in which hydrogen bonding and π-mediated interactions contribute substantially to binding affinity and molecular recognition [74].

The reliability of the docking protocol was further confirmed through redocking of the co-crystallized ligands into the active sites of the selected receptors. The resulting RMSD values ranged from 0.481 to 0.824 Å, remaining well below the widely accepted validation threshold of 2.0 Å. The lowest RMSD value was obtained for NAD(P)H oxidase (2CDU) (0.481 Å), followed by lanosterol 14-α-demethylase (4DKI) (0.689 Å) and penicillin-binding protein 2a (5TZ1) (0.824 Å). These results demonstrate an excellent reproduction of the experimental ligand conformations and confirm the robustness, reliability, and reproducibility of the docking methodology employed in this study.

Collectively, the molecular docking analyses revealed favorable ligand–target interactions and binding conformations that may contribute to the antioxidant and antimicrobial activities observed experimentally. Similar findings have been reported for terpenoids and other essential oil constituents, which frequently interact with proteins involved in oxidative stress regulation and microbial survival, supporting their biological potential [8,75]. Nevertheless, these results should be interpreted as mechanistic hypotheses that complement the experimental evidence rather than as direct proof of biological activity. Additional studies, including enzyme inhibition assays and molecular dynamics simulations, would be valuable to further validate the proposed interaction mechanisms and their biological relevance [12,76].

**Figure 6:**
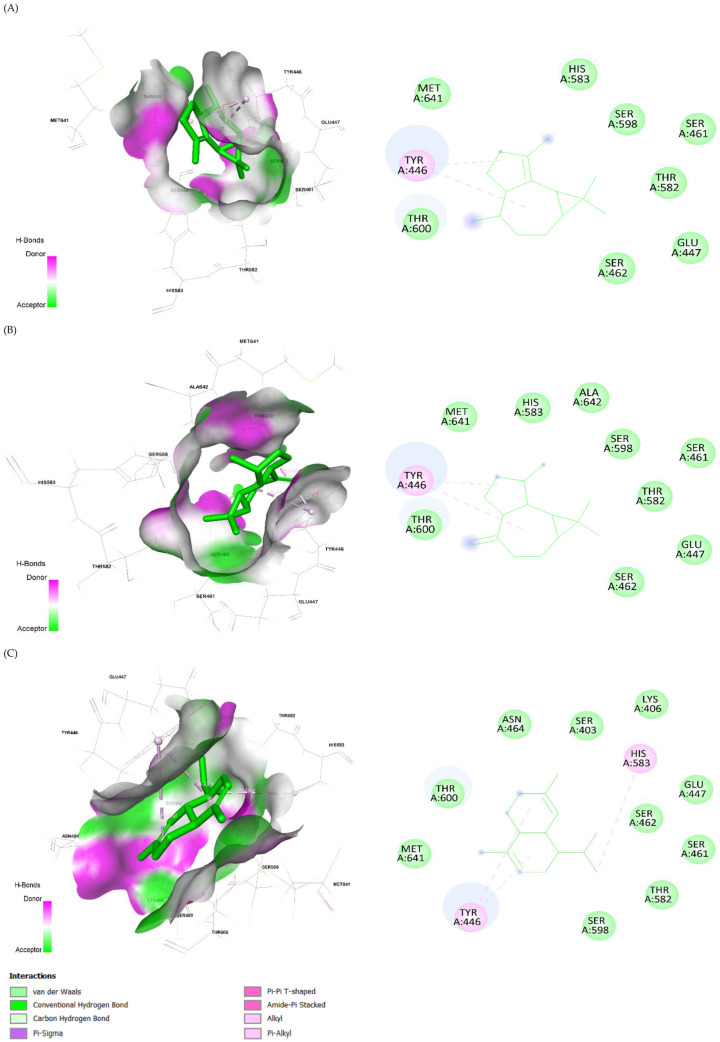
Two-dimensional (2D) and three-dimensional (3D) representations of the molecular interactions between three representative compounds, (**A**) α-gurjunene, (**B**) alloaromadendrene, and (**C**) 1,5-cadinadiene, and penicillin-binding protein 2a (PBP2a/MecA) from *Staphylococcus aureus* (PDB ID: 4DKI). The ligands are positioned within the binding pocket of the receptor and interact with several amino acid residues located in the active site, including Tyr446 and His583. The ligand–protein complexes are predominantly stabilized through hydrophobic contacts, particularly alkyl and π–alkyl interactions (pink dashed lines), which facilitate ligand accommodation within the active site. In addition, van der Waals forces (light green surfaces) contribute to the overall stability of the complexes. Regions associated with hydrogen-bond donor and acceptor properties are represented by magenta and green surfaces, respectively, illustrating the contribution of electrostatic interactions to ligand recognition and binding orientation.

**Figure 7:**
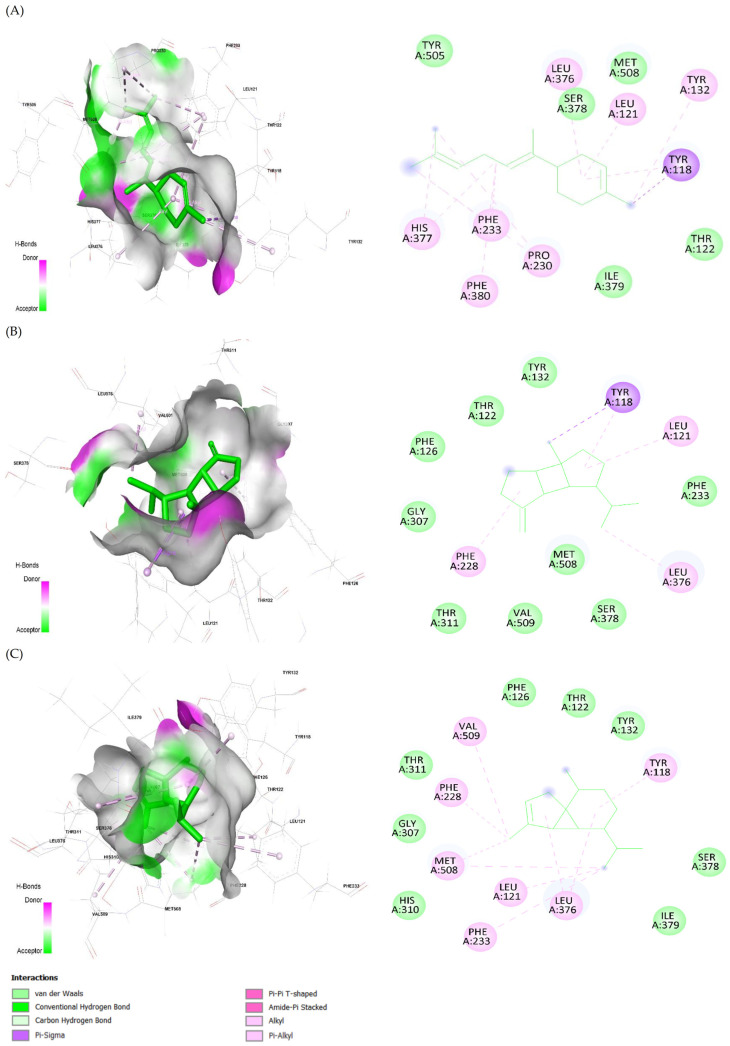
Two-dimensional (2D) and three-dimensional (3D) representations of the molecular interactions between three representative compounds, (**A**) trans-α-bisabolene, (**B**) (−)-β-bourbonene, and (**C**) α-cubebene, and sterol 14-α-demethylase (CYP51) from *Candida albicans* (PDB ID: 5TZ1). The ligands are accommodated within the hydrophobic binding cavity of the enzyme and interact with several amino acid residues, including Phe233, Tyr118, Leu121, and Met508. The ligand–protein complexes are mainly stabilized through hydrophobic interactions, particularly alkyl and π–alkyl contacts (pink and light purple dashed lines), which favor ligand accommodation within the active site. Additional stabilizing forces, such as π–π stacking interactions and van der Waals contacts (green surfaces), further contribute to complex stability. Moreover, polar interactions, including conventional hydrogen bonds and carbon–hydrogen bonds, are observed in specific binding orientations, supporting ligand recognition and the overall binding conformation.

**Figure 8:**
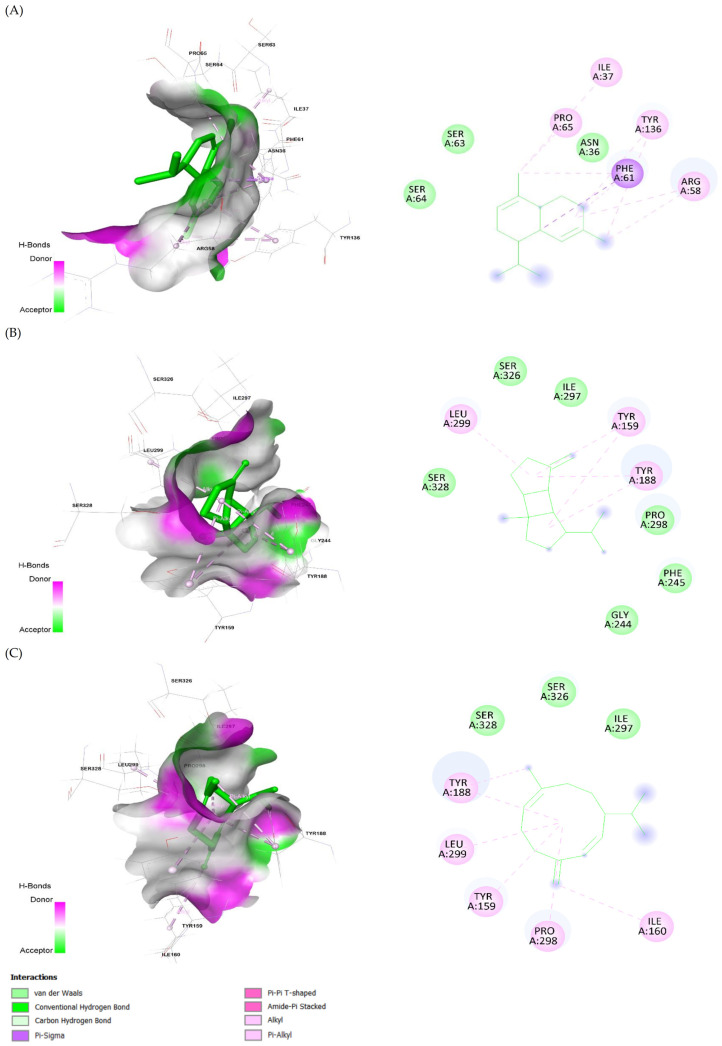
Two-dimensional (2D) and three-dimensional (3D) representations of the molecular interactions between three representative compounds, (**A**) 1,5-cadinadiene, (**B**) (−)-β-bourbonene, and (**C**) isogermacrene D, and NADPH oxidase from *Lactobacillus sanfranciscensis* (PDB ID: 2CDU). The ligands are located within the catalytic pocket of the enzyme, where they interact with several amino acid residues, including Tyr188, Leu299, Pro298, and Ile150. The ligand–protein complexes are predominantly stabilized by hydrophobic interactions, particularly alkyl and π–alkyl contacts (pink and light purple dashed lines), which promote proper accommodation of the ligands within the binding cavity. Additional stabilization is provided by van der Waals interactions (green surfaces), contributing to the overall integrity of the complexes. In certain binding conformations, weak polar interactions, such as carbon–hydrogen bonds, are also observed and may assist in maintaining ligand orientation within the active site.

For antibacterial activity, the best docking scores were obtained for α-gurjunene, alloaromadendrene and 1,5-cadinadiene with binding energies of −7.2, −7.1 and −7.1 kcal/mol, respectively. For antimycotic activity, the best docking scores were obtained for trans-α-bisabolene, (−)-ß-bourbonene and α-cubebene with binding energies of −8.0, −7.8 and −7.5 kcal/mol, respectively. For antioxidant activity, the best docking scores were obtained for 1,5-cadinadiene, (−)-ß-bourbonene and isogermacrene D with binding energies of −7.1, −7.0 and −6.9 kcal/mol, respectively (Table 5). These compounds met all the criteria for similarity to drugs; α-gurjunene showed hydrophobic interaction with Tyr446 for protein 4DKI (Figure 6); trans-α-bisabolene showed pi-sigma with Tyr118 and hydrophobic interactions with Leu121, Tyr132, Pro230, Phe233, Leu376, His377 and Phe380 for protein 5TZ1 (Figure 7); 1,5-cadinadiene showed pi-sigma with Phe61 and hydrophobic interactions with Ile37, Arg58, Pro65, and Tyr136 for protein 2CDU (Figure 8).

The binding affinity observed for the antibacterial molecules α-gurjunene (−7.2 kcal/mol) and alloaromadendrene (−7.1 kcal/mol) toward penicillin-binding protein 2a (PBP2a/MecA) (4DKI) suggests a potential inhibitory effect on bacterial cell wall biosynthesis, a key mechanism underlying resistance in methicillin-resistant *Staphylococcus aureus* (MRSA). This is consistent with previous studies on essential oils from the genus *Lippia*, which have demonstrated significant antibacterial activity associated with terpenoid constituents, particularly through interactions with membrane structures and intracellular targets involved in cell wall synthesis [2,23].

In addition, molecular docking studies have shown that plant-derived terpenoids can exhibit favorable binding affinities toward bacterial proteins, including PBP2a, with binding energies typically ranging from −6.0 to −8.5 kcal/mol, values indicative of stable ligand–protein interactions and potential biological activity [77]. Recent investigations on essential oil components have further demonstrated that sesquiterpenes can interact with bacterial enzymes and contribute to antimicrobial activity not only through direct protein binding but also via membrane disruption and synergistic effects [50,75,78].

Similarly, trans-α-bisabolene (−8.0 kcal/mol) and (−)-β-bourbonene (−7.8 kcal/mol) showed strong binding affinity toward sterol 14-α-demethylase (CYP51; 5TZ1), interacting with hydrophobic residues including Tyr118, Leu121, Phe228, and Leu376. Since CYP51 plays a key role in ergosterol biosynthesis, its inhibition compromises fungal membrane integrity, a mechanism commonly associated with antifungal activity. Recent docking studies have demonstrated that terpenoids and essential oil constituents can effectively interact with CYP51, supporting their potential as antifungal agents [79,80]. These findings are consistent with the antifungal activity observed in the present in vitro assays [81,82]. Moreover, binding energies ranging from −7.0 to −9.0 kcal/mol are generally considered indicative of stable ligand–enzyme interactions, in agreement with the affinities obtained in this study.

In the antioxidant context, the binding energies observed for 1,5-cadinadiene and (−)-β-bourbonene (−7.1 and −7.0 kcal/mol, respectively) against NADPH oxidase (2CDU) suggest a potential mechanism involving modulation of reactive oxygen species (ROS) production. NADPH oxidase is a key enzymatic source of oxidative stress, and its inhibition has been associated with antioxidant effects. Recent in silico studies have demonstrated that essential oil components can interact with NADPH oxidase, supporting their role in reducing oxidative damage through enzyme modulation [83] a result consistent with findings reported by Moura et al. [84], where the biological activity of *Lippia* essential oil was associated with its terpenoid composition, particularly thymol, which exhibited antioxidant and neuroprotective effects.

The interaction patterns identified in this study suggest that the volatile constituents of *Lippia alba* may exhibit a multitarget mode of action, as evidenced by their favorable interactions with proteins associated with oxidative stress, bacterial resistance, and fungal metabolism. Such behavior is consistent with previous reports indicating that terpenoids and other essential oil constituents can modulate multiple biological pathways, thereby contributing to their overall pharmacological potential [75,85]. In this context, the docking results provide a plausible mechanistic basis for the antioxidant, antibacterial, and antifungal activities observed experimentally.

To date, relatively few studies have investigated the molecular interactions of *L. alba* volatile constituents with biological targets related to antioxidant and antimicrobial activities. Therefore, the present work contributes additional evidence by integrating chemical characterization, biological assays, and molecular docking analyses to explore potential mechanisms underlying the observed bioactivities. The consistency between the experimental and computational findings supports the hypothesis that the biological effects of EOLA may arise from the combined action of multiple constituents rather than from a single dominant compound.

Although molecular docking provided valuable insights into ligand–target recognition, it represents a static model of molecular interactions and should be interpreted with caution. Future studies incorporating molecular dynamics simulations and binding free-energy calculations would help evaluate the stability, flexibility, and persistence of the predicted complexes under more physiologically relevant conditions, thereby providing a deeper understanding of the molecular mechanisms involved.

The present findings should be interpreted within the broader context of the extensive literature available for *L. alba*. Rather than establishing novel biological activities for the species, this study provides additional evidence regarding the chemical composition and biological potential of a Peruvian carvone-rich chemotype. The integration of ethnobotanical information, in vitro bioassays, and molecular docking analyses offers a comprehensive perspective on the pharmacological relevance of this population and highlights the importance of geographical origin as a factor influencing the chemical and biological variability of medicinal plants. A summary of the main ethnobotanical, phytochemical, biological, and molecular docking findings is provided in Table 6.

Beyond its demonstrated biological activities, the chemical profile and antimicrobial performance of EOLA suggest promising opportunities for its incorporation into phytopharmaceutical and nutraceutical formulations. The pronounced antibacterial and antifungal activities observed in the present study, particularly at higher concentrations, support its potential application in topical preparations such as creams, gels, ointments, wound dressings, mouthwashes, and antiseptic formulations. In addition, the antioxidant properties of the oil may be advantageous for the development of natural preservative systems and functional ingredients in food, cosmetic, and pharmaceutical products. Recent advances in essential oil technology have also highlighted nanoemulsions, liposomes, solid lipid nanoparticles, and polymeric encapsulation systems as effective strategies to improve stability, solubility, bioavailability, and controlled release of volatile constituents, thereby enhancing their biological efficacy.

Despite these prospects, several challenges must be addressed before large-scale industrial application can be achieved. Essential oils are inherently susceptible to oxidation, volatilization, and degradation during storage and processing, which may affect product stability and reproducibility. Furthermore, the pronounced chemotypic variability of *Lippia alba*, influenced by genetic, environmental, seasonal, and agronomic factors, can lead to significant fluctuations in essential oil composition and biological activity. Consequently, standardization of raw materials, cultivation practices, harvesting conditions, and extraction procedures is essential to ensure batch-to-batch consistency. Additional considerations include optimization of extraction yield, economic feasibility, regulatory approval, safety assessment, and validation through in vivo and clinical studies. Therefore, while the results obtained support the potential of EOLA as a source of bioactive ingredients, further technological and pharmacological investigations are required to facilitate its translation into commercially viable formulations.

## 3. Materials and Methods

### 3.1. Chemicals

Ethanol was obtained from Spectrum Chemical (New Brunswick, NJ, USA), while distilled water was supplied by Dropaksa (Lima, Peru). The reagents 2,2-diphenyl-1-picrylhydrazyl (DPPH), 2,2′-azinobis(3-ethylbenzothiazoline-6-sulfonic acid) diammonium salt (ABTS), potassium persulfate, Trolox (6-hydroxy-2,5,7,8-tetramethylchroman-2-carboxylic acid), fluconazole, oxacillin, sodium chloride, dimethyl sulfoxide (DMSO), and Mueller-Hinton agar were purchased from Sigma-Aldrich (St. Louis, MO, USA). Sabouraud dextrose agar was obtained from Merck (Darmstadt, Germany). All chemicals, reagents, and solvents used throughout the study were of analytical grade.

### 3.2. Ethnobotanical Study

An ethnobotanical survey was conducted in wholesale markets located in Trujillo, La Libertad, Peru, involving 64 medicinal plant vendors selected through random sampling. Data was collected through semi-structured interviews administered by trained researchers after obtaining written informed consent from all participants. The survey gathered information on vernacular plant names, traditional medicinal uses, methods of preparation and administration, plant parts utilized, health conditions treated, combinations with other medicinal species, dosage practices, and sources of traditional knowledge. The study design and data collection procedures were based on a previously reported ethnobotanical methodology [86]. Ethical approval for the study was obtained from the Ethics Committee of Norbert Wiener Private University (approval code: A0056-2025; approved on 13 October 2025).

### 3.3. Plant Material

The species studied was *Lippia alba* (Mill.) N.E. Br. ex Britton & P. Wilson. Plant material was collected on 3 November 2025 in the Agallpampa District, Otuzco Province, La Libertad Region, Peru (7°57′08″ S, 78°31′00″ W; 2927 m a.s.l.), during the full flowering stage (anthesis). Sampling was performed during the dry season, a period characterized by high solar radiation and limited water availability in the Andean region, conditions that may favor the accumulation of secondary metabolites. A voucher specimen was prepared following the standard procedures of the *Herbarium Truxillense* (HUT), where it was taxonomically identified and deposited under accession number 60824.

### 3.4. Essential Oil Extraction

The essential oil of *Lippia alba* (EOLA) was obtained by hydrodistillation using a 6 L Clevenger-type apparatus. Fresh leaves collected during the flowering stage on 24 November 2025 were air-dried prior to extraction. From an initial 6.0 kg of fresh plant material, 2.0 kg of dried biomass were obtained. Hydrodistillation was performed in triplicate using three independent batches of 410.2 g of dried leaves, with a plant-to-water ratio of 1:8 (*w*/*v*) and an extraction time of 3 h. The volumes of essential oil recovered in each extraction were 0.89, 0.95, and 1.12 mL, corresponding to an average yield of 0.240 ± 0.027% (*v*/*w*) calculated on a dry weight basis. The obtained essential oil was dehydrated with anhydrous sodium sulfate (Na_2_SO_4_) and stored in amber vials at 4 °C until further analysis. The residual aqueous fraction containing non-volatile constituents was also collected and preserved in amber containers at 4 °C for subsequent analyses.

### 3.5. Identification of Volatile Components

The chemical composition of *Lippia alba* essential oil (EOLA) was analyzed by gas chromatography coupled to flame ionization detection (GC–FID) and gas chromatography–mass spectrometry (GC–MS) using Shimadzu instruments (Shimadzu Corporation, Kyoto, Japan). Both analyses were performed under identical chromatographic conditions to ensure consistency in compound identification and relative quantification. Separation was achieved on an HP-5MS capillary column (5% phenyl–95% dimethylpolysiloxane; 30 m × 0.25 mm i.d.; film thickness 0.25 μm). Nitrogen was used as the carrier gas at a flow rate of 46.5 mL/min. The oven temperature program and operating conditions were optimized for the separation of volatile constituents. The injector, oven, and detector temperatures were maintained at 280 °C, 280 °C, and 290 °C, respectively. For GC–FID analysis, 0.2 μL of undiluted essential oil was injected in split mode with a split ratio of 1:10, whereas GC–MS analysis was conducted using a split ratio of 1:100. For GC–MS, the injector, ion source, and interface temperatures were set at 280 °C, 230 °C, and 270 °C, respectively. Mass spectra were acquired in electron ionization (EI) mode at 70 eV over a mass range of 35–650 *m*/*z*. Compound identification was based on the comparison of mass spectra with those contained in the NIST (version 4.1) and Wiley (7th edition) spectral libraries. Identification was further confirmed by calculating retention indices (RI) relative to a homologous series of *n*-alkanes (C_9_–C_31_) and comparing the obtained values with those reported in the literature by Adams [87]. In addition, the characteristic fragmentation patterns of the detected compounds were examined and compared with published data to enhance the accuracy and reliability of metabolite identification.

### 3.6. Antioxidant Activity Evaluation

#### 3.6.1. DPPH Radical Scavenging Activity

The antioxidant activity of *Lippia alba* essential oil (EOLA) was evaluated using the DPPH (2,2-diphenyl-1-picrylhydrazyl) radical scavenging assay with minor modifications. A 0.1 mM DPPH solution was prepared in 96% ethanol. Briefly, 100 μL of EOLA diluted in 0.3% DMSO was mixed with the DPPH solution in a 10 mL volumetric flask, homogenized, and incubated in the dark at room temperature for 30 min. Trolox was used as the reference antioxidant standard at concentrations ranging from 3 to 30 μM to construct the calibration curve. Following incubation, absorbance was measured at 517 nm using a C7000V UV–Vis spectrophotometer (Peak Instruments, Houston, TX, USA). All determinations were performed in triplicate. Antioxidant activity was calculated from the Trolox calibration curve and expressed as milligrams of Trolox equivalents per gram of essential oil (mg TE/g EOLA) [88].

#### 3.6.2. ABTS Radical Scavenging Activity

The antioxidant activity of *Lippia alba* essential oil (EOLA) was evaluated using the ABTS [2,2′-azino-bis(3-ethylbenzothiazoline-6-sulfonic acid)] radical cation decolorization assay with minor modifications. The ABTS stock solution was prepared by mixing equal volumes of ABTS (7 mM) and potassium persulfate (K_2_S_2_O_8_, 2.45 mM), followed by incubation in the dark at room temperature for 16 h. Prior to analysis, the resulting solution was diluted with 50% ethanol to obtain the working solution. Briefly, 100 μL of EOLA diluted in 0.3% DMSO was transferred to a 10 mL volumetric flask containing the ABTS working solution. The mixture was gently homogenized and incubated in the dark at room temperature for 30 min. Trolox was used as the reference antioxidant standard at concentrations ranging from 3 to 20 μM for calibration curve construction. After incubation, absorbance was measured at 734 nm using a C7000V UV–Vis spectrophotometer (Peak Instruments, Houston, TX, USA). All determinations were performed in triplicate. Antioxidant activity was calculated from the Trolox calibration curve and expressed as milligrams of Trolox equivalents per gram of essential oil (mg TE/g EOLA) [89].

### 3.7. Antifungal Activity

The antifungal activity of the essential oil of *Lippia alba* (EOLA) was evaluated against *Candida albicans* ATCC 10231 using a disk diffusion assay adapted from CLSI M44 guidelines [18,90,91,92]. The fungal strain was subcultured on Sabouraud Dextrose Agar (SDA) and incubated at 35 ± 2 °C for 18–24 h. Fresh colonies were suspended in sterile 0.85% saline solution and adjusted to a 0.5 McFarland standard (≈1–5 × 10^6^ CFU/mL) according to CLSI recommendations for yeast susceptibility testing [18,90,91,92].

EOLA was diluted in dimethyl sulfoxide (DMSO) to obtain final concentrations of 25%, 50%, and 75% (*v*/*v*). DMSO at 2% (*v*/*v*) was used as the negative control, whereas fluconazole (25 µg/disk) served as the positive control. DMSO was selected because of its ability to solubilize hydrophobic essential oils while exhibiting negligible antifungal activity at low concentrations [93,94,95]. Sterile 6-mm paper disks were impregnated with 20 µL of each treatment, briefly dried under sterile airflow, and immediately placed onto the inoculated agar surface.

Mueller–Hinton agar supplemented for yeast diffusion or Sabouraud-type agar was uniformly inoculated with the standardized *C. albicans* suspension using sterile swabs [90,92]. Subsequently, disks containing EOLA, DMSO, and fluconazole were placed equidistantly on the agar plates, which were incubated in an inverted position at 35 ± 2 °C for 24 h. Antifungal activity was determined by measuring inhibition-zone diameters (mm), including the 6-mm disk diameter, using a digital caliper with 0.1-mm precision. Results were expressed as mean ± standard deviation of three independent replicates [18,91]. Since essential oils generally exhibit limited diffusion in agar media, the assay was considered a qualitative to semi-quantitative screening method [62,96].

Quality control was performed in each experimental run using *C. albicans* ATCC 10231 and fluconazole disks according to CLSI M44 criteria [18,62]. The DMSO control confirmed the absence of solvent interference, and fresh EOLA dilutions were prepared before each assay to minimize volatilization of volatile constituents.

### 3.8. Antibacterial Activity

The antibacterial activity of the essential oil of *Lippia alba* (EOLA) was evaluated against *Staphylococcus aureus* ATCC 25923 using a disk diffusion assay based on CLSI recommendations [91,93,97,98,99]. The bacterial strain was used as both the test organism and quality-control strain. Cultures were subcultured twice on Mueller–Hinton agar (MHA) and incubated at 35–37 °C for 18–24 h. Fresh colonies were suspended in sterile saline solution and adjusted to a 0.5 McFarland standard (≈1 × 10^8^ CFU/mL) according to CLSI guidelines for disk diffusion susceptibility testing [91,93,97,98].

EOLA was diluted in dimethyl sulfoxide (DMSO) to final concentrations of 25%, 50%, and 75% (*v*/*v*). DMSO at 2% (*v*/*v*) was used as the negative control, whereas doxycycline disks (30 µg/disk) served as the positive control. DMSO was selected because of its effectiveness in solubilizing hydrophobic essential oils while exhibiting negligible antibacterial activity at concentrations ≤ 2% (*v*/*v*); therefore, a 2% DMSO vehicle control was included to verify the absence of solvent-mediated inhibition.

Mueller-Hinton agar plates were uniformly inoculated with the standardized *S. aureus* suspension using sterile swabs. Subsequently, sterile 6-mm paper disks impregnated with 20 µL of each treatment were briefly dried under sterile conditions and placed onto the agar surface. Doxycycline disks were applied according to the manufacturer’s instructions. Plates were incubated at 35 ± 2 °C for 18–24 h, after which inhibition-zone diameters (mm), including the disk diameter, were measured using a digital caliper with 0.1-mm precision. Results were expressed as mean ± standard deviation of three independent replicates. Due to the limited diffusion capacity of essential oils in agar media, inhibition zones were interpreted comparatively rather than according to clinical breakpoints [99].

Quality control was performed using *S. aureus* ATCC 25923 and doxycycline control disks following CLSI M02 criteria. The DMSO control confirmed the absence of solvent interference, and assays not meeting quality-control requirements were repeated.

### 3.9. ADMET In Silico Prediction

The physicochemical properties of the 27 volatile compounds identified in EOLA were initially evaluated according to Lipinski’s Rule of Five [100]. Subsequently, their absorption, distribution, metabolism, excretion, and toxicity (ADMET) profiles were predicted using the SwissADME (http://www.swissadme.ch; accessed 25 January 2026) and pkCSM (http://biosig.unimelb.edu.au/pkcsm; accessed 16 February 2026) web platforms [101,102]. In addition, the BOILED-Egg model was employed to assess passive gastrointestinal absorption and blood–brain barrier (BBB) permeability, allowing the identification of compounds with potential central nervous system (CNS) accessibility [103].

### 3.10. Molecular Docking Simulation

Molecular docking simulations were performed to investigate the potential interactions between the volatile constituents of EOLA and protein targets associated with the biological activities evaluated experimentally. Docking calculations were carried out using AutoDock Vina v1.2.1 [104]. The three-dimensional crystal structures of penicillin-binding protein 2a (PBP2a/MecA; PDB ID: 5TZ1), lanosterol 14-α-demethylase (CYP51; PDB ID: 4DKI), and NAD(P)H oxidase (PDB ID: 2CDU) were retrieved from the RCSB Protein Data Bank (https://www.rcsb.org/) [105].

The selected targets were chosen based on their biological relevance to the antioxidant, antifungal, and antibacterial activities assessed in this study. NAD(P)H oxidase (2CDU), a key enzyme involved in reactive oxygen species (ROS) production and oxidative stress pathways, was used as the antioxidant target. Lanosterol 14-α-demethylase (4DKI), an essential enzyme in ergosterol biosynthesis and fungal membrane maintenance, was selected as the antifungal target. Penicillin-binding protein 2a (5TZ1), which plays a central role in bacterial cell wall synthesis and antimicrobial resistance in *Staphylococcus aureus*, was employed as the antibacterial target.

Protein preparation was performed using UCSF Chimera v1.18, including the removal of co-crystallized ligands, ions, and water molecules. Energy minimization was subsequently carried out using Swiss-PdbViewer v4.1 [106]. The prepared receptors were parameterized by adding polar hydrogen atoms and Gasteiger charges. Three-dimensional structures of all ligands were obtained from the PubChem database (https://pubchem.ncbi.nlm.nih.gov/) [107]. Ascorbic acid, fluconazole, and oxacillin were used as reference ligands for antioxidant, antifungal, and antibacterial analyses, respectively. Ligand geometries were optimized using the MMFF94 force field implemented in Avogadro v1.2.

Docking simulations were conducted for all 27 volatile compounds identified in EOLA. Grid boxes were defined around the active sites of each receptor. For PBP2a/MecA (5TZ1), a 25 × 25 × 25 Å grid was centered at X = 27.25, Y = 28.12, and Z = 86.46. For CYP51 (4DKI), a 25 × 25 × 25 Å grid was centered at X = 67.84, Y = 70.77, and Z = 4.60. For NAD(P)H oxidase (2CDU), a 30 × 30 × 30 Å grid was centered at X = 2.44, Y = 2.14, and Z = −1.08.

The docking protocol was validated through a redocking procedure. Briefly, the co-crystallized ligand was extracted from each experimental structure and subsequently redocked into its original binding site using the same docking parameters applied in the main simulations. Receptor preparation was performed using the Dock Prep module of UCSF Chimera with default settings, including bond order assignment, hydrogen addition, hydrogen-bond network optimization, and restrained energy minimization. Validation was assessed by calculating the root-mean-square deviation (RMSD) between the crystallographic and redocked ligand conformations, considering only heavy atoms. RMSD values ≤ 2.0 Å were considered indicative of successful protocol validation and reliable reproduction of the experimental binding mode [108].

The best docking poses were selected according to the lowest binding energy values obtained. The resulting ligand–protein complexes were visualized using UCSF Chimera v1.18 [109], while two-dimensional (2D) and three-dimensional (3D) interaction profiles were generated and analyzed using Discovery Studio Visualizer v2025 [110].

### 3.11. Statistical Analysis

All experiments were performed in triplicate, and results are presented as mean ± standard deviation (SD) (*n* = 3). Data distribution and homogeneity of variances were evaluated using the Shapiro–Wilk and Levene’s tests, respectively. When the assumptions of normality and homoscedasticity were met, differences among groups were analyzed by one-way analysis of variance (ANOVA) followed by Tukey’s honestly significant difference (HSD) post hoc test. For data that did not meet these assumptions, the Kruskal–Wallis test followed by Dunn’s multiple-comparison test was applied. Statistical significance was established at *p* < 0.05. All statistical analyses were performed using GraphPad Prism version 11.0 (GraphPad Software, San Diego, CA, USA).

## 4. Conclusions

The present study provides a comprehensive characterization of a Peruvian carvone-rich chemotype of *Lippia alba* through the integration of ethnobotanical information, chemical profiling, biological evaluation, and molecular docking analyses. GC–MS analysis revealed a volatile profile dominated by oxygenated monoterpenes, particularly (−)-carvone and dihydrocarvyl acetate, which are likely associated with the biological activities observed. The essential oil exhibited antioxidant activity together with notable antifungal and antibacterial effects against *Candida albicans* and *Staphylococcus aureus*, respectively. These findings support the ethnopharmacological relevance of *L. alba* and confirm the biological potential of the carvone chemotype as a source of bioactive natural products. Furthermore, molecular docking analyses identified favorable interactions between the volatile constituents and protein targets associated with oxidative stress and microbial survival, providing mechanistic support for experimental observations. Overall, the integration of traditional knowledge, phytochemical characterization, in vitro bioassays, and computational approaches provides a comprehensive understanding of the biological potential of *L. alba* essential oil. These findings contribute to the growing body of evidence supporting the value of *L. alba* as a promising source of multifunctional phytochemicals with potential applications in pharmaceutical, cosmetic, and natural antimicrobial formulations.

Several limitations of the present study should be acknowledged. First, a significant methodological limitation of the present study is the reliance on the agar disk diffusion method. Given the poor aqueous diffusion of lipophilic matrices such as essential oils, this assay likely underestimates the true antimicrobial potency of EOLA. More importantly, the lack of Minimum Inhibitory Concentration (MIC) and Minimum Bactericidal/Fungicidal Concentration (MBC/MFC) determinations leaves the therapeutic margin and safety profile of the essential oil unexplored. Future investigations must incorporate broth microdilution assays and in vivo toxicity models to accurately establish these critical pharmacological parameters prior to any clinical or commercial application.

Second, biological activities were assessed exclusively under in vitro conditions. Consequently, the pharmacokinetic behavior, efficacy, and safety of the essential oil under physiological conditions remain unknown. In vivo studies, toxicity assessments, and formulation development will be necessary to establish its therapeutic potential and practical applicability.

Third, although molecular docking provided valuable mechanistic insights, it represents a static predictive approach that does not account for protein flexibility, solvent effects, or the dynamic nature of ligand–protein interactions. Future investigations employing molecular dynamics simulations and binding free-energy calculations would help validate the stability and biological relevance of the predicted complexes.

Finally, the ethnobotanical survey was conducted exclusively with herbal vendors from Trujillo, northern Peru, and therefore reflects traditional knowledge within a specific geographical and cultural context. The unequal representation of male and female participants and the absence of quantitative ethnobotanical indices, such as Use Value (UV), Fidelity Level (FL), Relative Frequency of Citation (RFC), and Informant Consensus Factor (ICF), may limit the generalizability of the findings. Future studies should include larger and geographically diverse populations together with standardized ethnobotanical metrics to better assess the cultural significance and medicinal relevance of *L. alba* across different regions.

Despite these limitations, the present work provides an integrated framework linking traditional use, chemical composition, biological activity, and potential molecular mechanisms, thereby establishing a solid foundation for future pharmacological and phytopharmaceutical research on *Lippia alba* essential oil.

## Figures and Tables

**Table 1:** Antioxidant activity of *Lippia alba* essential oil (EOLA) determined by DPPH and ABTS assays.

Sample	DPPH (mg TE/g)	ABTS (mg TE/g)
EOLA	24.10 ± 0.10	34.53 ± 0.11

Values are expressed as mean ± standard deviation (SD) of three independent determinations (*n =* 3); EOLA: essential oil of *Lippia alba*; TE: Trolox equivalents.

**Table 2:** GC–MS characterization of volatile compounds present in *Lippia alba* essential oil (EOLA).

N.	Peak Name ^1,2^	RI ^3^	RI Lit ^4^	Relative Area (%)
1	α-pinene	939	939	0.11
2	Camphene	953	953	0.04
3	Sabinene	940	969	0.04
4	ß-pinene	980	979	2.12
5	α-thujene	930	924	2.46
6	Dihydrocarvyl acetate	1330	1318	17.74
7	Terpinolene	1020	1087	1.16
8	Linalool	1090	1096	1.92
9	(−)-carvone	1245	1243	63.79
10	Piperitenone	1350	1338	0.38
11	α-cubebene	1345	1348	3.40
12	(−)-ß-bourbonene	1383	1384	0.03
13	α-gurjunene	1390	1498	2.94
14	ß-copaene	1425	1468	0.08
15	Isogermacrene D	1450	1508	0.12
16	Seychellene	1480	1458	0.06
17	Alloaromadendrene	1500	1462	0.38
18	α-muurolene	1510	1479	0.03
19	1,5-cadinadiene	1530	1510	0.15
20	Sesquiterpene alcohol derivative	1545	1588	0.36
21	Trans- α-Bisabolene	1560	1382	0.01
22	Isoaromadendrene epoxide	1589	1590	0.07
23	Salvialene derivative	1600	1690	0.04
24	(−)-spathulenol	1615	1577	0.04
25	Tricyclic sesquiterpene alcohol	1630	1668	0.02
26	Ylangenal	1675	1805	0.05
27	Sesquiterpene alcohol derivative	1700	1650	0.12
Hydrocarbon monoterpenes				5.93%
Oxygenated monoterpenes				83.83%
Sesquiterpenes				7.90%

^1^ Elution order on the HP-5MS column. ^2^ Compound identification was based on retention indices (RI) and mass spectral data (MS). ^3^ RI values were calculated using a homologous series of *n*-alkanes (C_9_–C_31_). ^4^ Experimental RI values were compared with literature data reported by Adams and verified through spectral matching with the NIST (v4.1) and Wiley (7th edition) libraries. Compound assignments should be considered tentative.

**Table 3:** Predicted molecular and physicochemical properties of the volatile constituents identified in EOLA.

N.	Compounds	MW ≤500 Da	HBD ≤5	HBA ≤10	cLog P_o/w_ ≤5	MR 40–130	N° Violations ≤2	Drug-Likeness
1	α-pinene	Yes	Yes	Yes	No	Yes	1	Yes
2	Camphene	Yes	Yes	Yes	No	Yes	1	Yes
3	Sabinene	Yes	Yes	Yes	No	Yes	1	Yes
4	ß-pinene	Yes	Yes	Yes	No	Yes	1	Yes
5	α-thujene	Yes	Yes	Yes	No	Yes	1	Yes
6	Dihydrocarvyl acetate	Yes	Yes	Yes	Yes	Yes	0	Yes
7	Terpinolene	Yes	Yes	Yes	Yes	Yes	0	Yes
8	Linalool	Yes	Yes	Yes	Yes	Yes	0	Yes
9	(−)-carvone	Yes	Yes	Yes	Yes	Yes	0	Yes
10	Piperitenone	Yes	Yes	Yes	Yes	Yes	0	Yes
11	α-cubebene	Yes	Yes	Yes	No	Yes	1	Yes
12	(−)-ß-bourbonene	Yes	Yes	Yes	No	Yes	1	Yes
13	α-gurjunene	Yes	Yes	Yes	No	Yes	1	Yes
14	ß-copaene	Yes	Yes	Yes	No	Yes	1	Yes
15	Isogermacrene D	Yes	Yes	Yes	No	Yes	1	Yes
16	Seychellene	Yes	Yes	Yes	No	Yes	1	Yes
17	Alloaromadendrene	Yes	Yes	Yes	No	Yes	1	Yes
18	α-muurolene	Yes	Yes	Yes	No	Yes	1	Yes
19	1,5-cadinadiene	Yes	Yes	Yes	No	Yes	1	Yes
20	Sesquiterpene alcohol derivative	Yes	Yes	Yes	Yes	Yes	0	Yes
21	Trans-α-Bisabolene	Yes	Yes	Yes	No	Yes	1	Yes
22	Isoaromadendrene epoxide	Yes	Yes	Yes	Yes	Yes	0	Yes
23	Salvialene derivative	Yes	Yes	Yes	Yes	Yes	0	Yes
24	(−)-spathulenol	Yes	Yes	Yes	Yes	Yes	0	Yes
25	Tricyclic sesquiterpene alcohol	Yes	Yes	Yes	Yes	Yes	0	Yes
26	Ylangenal							
27	Sesquiterpene alcohol derivative	Yes	Yes	Yes	Yes	Yes	0	Yes

**Table 4:** Predicted absorption, distribution, metabolism, excretion, and toxicity (ADMET) parameters of the volatile compounds present in EOLA.

N.	Compounds	Absorption	Distribution		Metabolism							Excretion	Toxicity		
		Intestinal Absorption (Human)			Substrate Inhibitor							Total Clearance	AMES Toxicity	Hepatotoxicity	Skin Sensitization
					Cytochromes										
			BHE Permeability	CNS Permeability	2D6	3A4	1A2	2C19	2C9	2D6	3A4				
		(% Absorbed)	(Log BB)	(Log PS)	Categorical (Yes/No)							(Log mL/min/kg)	Categorical (Yes/No)		
1	α-pinene	96.041	0.791	−2.201	No	No	No	No	No	No	No	0.043	No	No	No
2	Camphene	94.148	0.787	−1.71	No	No	No	No	No	No	No	0.049	No	No	No
3	Sabinene	95.356	0.836	−1.463	No	No	No	No	No	No	No	0.071	No	No	No
4	ß-pinene	95.525	0.818	−1.857	No	No	No	No	No	No	No	0.03	No	No	No
5	α-thujene	95.256	0.81	−1.793	No	No	No	No	No	No	No	0.077	No	No	No
6	Dihydrocarvyl acetate	96.692	0.523	−2.695	No	No	No	No	No	No	No	1.322	No	No	Yes
7	Terpinolene	95.6	0.695	−2.317	No	No	No	No	No	No	No	0.218	No	No	Yes
8	Linalool	93.163	0.598	−2.339	No	No	No	No	No	No	No	0.446	No	No	Yes
9	(−)-carvone	97.702	0.588	−2.478	No	No	No	No	No	No	No	0.225	No	No	Yes
10	Piperitenone	96.972	0.557	−2.418	No	No	No	No	No	No	No	0.156	No	No	Yes
11	α-cubebene	95.964	0.86	−1.552	No	Yes	Yes	No	No	No	No	0.98	No	No	No
12	(−)-ß-bourbonene	95.668	0.879	−1.218	No	Yes	Yes	No	No	No	No	0.967	Yes	No	No
13	α-gurjunene	96.566	0.792	−2.136	No	No	No	No	Yes	No	No	0.907	No	No	No
14	ß-copaene	95.906	0.905	−1.335	No	Yes	Yes	No	No	No	No	0.962	No	No	No
15	Isogermacrene D	95.59	0.723	−2.138	No	No	No	No	No	No	No	1.42	No	No	Yes
16	Seychellene	96.161	0.866	−1.606	No	Yes	Yes	No	No	No	No	0.983	No	No	No
17	Alloaromadendrene	95.302	0.822	−1.769	No	Yes	No	No	No	No	No	0.926	No	No	No
18	α-muurolene	94.64	0.785	−1.895	No	No	No	No	No	No	No	1.18	No	No	Yes
19	1,5-cadinadiene	94.64	0.785	−1.895	No	No	No	No	No	No	No	1.18	No	No	Yes
20	Sesquiterpene alcohol derivative	94.206	0.669	−1.768	No	Yes	No	No	No	No	No	0.885	No	No	Yes
21	Trans- α-Bisabolene	94.345	0.767	−2.057	No	No	No	No	No	No	No	1.437	No	No	Yes
22	Isoaromadendrene epoxide	95.805	0.732	−2.114	No	Yes	No	No	No	No	No	0.753	No	No	Yes
23	Salvialene derivative	96.482	0.757	−1.902	No	Yes	Yes	No	No	No	No	1.034	No	No	Yes
24	(−)-spathulenol	93.906	0.617	−2.526	No	No	No	Yes	Yes	No	No	0.895	No	No	Yes
25	Tricyclic sesquiterpene alcohol	92.706	0.622	−2.186	No	Yes	Yes	No	No	No	No	0.809	No	No	Yes
26	Ylangenal	96.231	0.759	−1.75	No	Yes	Yes	No	No	No	No	−0.092	No	No	Yes
27	Sesquiterpene alcohol derivative	94.206	0.669	−1.768	No	Yes	No	No	No	No	No	0.885	No	No	Yes

**Table 5:** Docking scores (kcal/mol) of the volatile compounds identified in EOLA and the corresponding reference ligands against the selected molecular targets.

N.	ID PubChem	Compounds	Antibacterial PDB: 5TZ1	Antifungal PDB: 4DKI	Antioxidant PDB: 2CDU
1	6654	α-pinene	−5.1	−5.9	−5.2
2	6616	Camphene	−5.0	−5.6	−5.3
3	10887971	Sabinene	−4.7	−5.8	−5.3
4	14896	ß-pinene	−4.8	−5.8	−5.1
5	17868	α-thujene	−4.8	−5.3	−5.4
6	30248	Dihydrocarvyl acetate	−6.0	−6.2	−6.0
7	11463	Terpinolene	−5.5	−6.5	−6.1
8	6549	Linalool	−4.9	−5.9	−5.1
9	439570	(−)-carvone	−5.6	−6.3	−5.8
10	381152	Piperitenone	−5.6	−6.2	−5.9
11	442359	α-cubebene	−6.8	−7.5	−6.7
12	62566	(−)-ß-bourbonene	−7.0	−7.8	−7.0
13	521243	α-gurjunene	−7.2	−6.8	−6.7
14	87529	ß-copaene	−6.7	−7.4	−6.7
15	91723653	Isogermacrene D	−6.7	−7.2	−6.9
16	22211634	Seychellene	−5.7	−7.2	−6.1
17	10899740	Alloaromadendrene	−7.1	−7.0	−6.8
18	12306047	α-muurolene	−7.1	−7.1	−6.6
19	101708	1,5-cadinadiene	−7.1	−7.1	−7.1
20	11976203	Sesquiterpene alcohol derivative	−6.9	−6.9	−6.7
21	24798703	Trans-α-Bisabolene	−6.2	−8.0	−6.8
22	534398	Isoaromadendrene epoxide	−6.6	−7.2	−6.7
23	85669481	Salvialene derivative	−6.6	−7.3	−6.5
24	13854255	(−)-spathulenol	−6.8	−7.2	−6.5
25	573804	Tricyclic sesquiterpene alcohol	−3.0	−3.8	−3.4
26	12303906	Ylangenal	−6.7	−7.4	−6.8
27	13304974	Sesquiterpene alcohol derivative	−6.8	−7.4	−6.5
28	6196	Oxacilina	−8.6		
29	3365	Fluconazol		−6.9	
30	54670067	Vitamina C			−6.2

**Table 6:** Summary of the main ethnobotanical, phytochemical, biological, and molecular docking findings of *Lippia alba* essential oil.

Category	Main Findings
Ethnobotanical relevance	Principal traditional uses: menstrual regulation (33.3%), relaxant (16.7%), digestive (16.7%), and treatment of culturally defined conditions (16.7%).
Major volatile constituents	(−)-Carvone (63.79%), dihydrocarvyl acetate (17.74%), α-cubebene (3.40%), α-gurjunene (2.94%), β-pinene (2.12%), linalool (1.92%), and terpinolene (1.16%).
Chemotype	Carvone chemotype characterized by a predominance of oxygenated monoterpenes (83.83%).
Antioxidant activity	DPPH: 24.10 ± 0.10 mg TE/g EO; ABTS: 34.53 ± 0.11 mg TE/g EO.
Antifungal activity	Candida albicans: inhibition zone of 21.92 mm at EO 75%, approaching the activity of fluconazole (25.98 mm).
Antibacterial activity	Staphylococcus aureus: inhibition zone of 20.67 mm at EO 75%, exceeding the activity of doxycycline (19.75 mm).
Best antioxidant docking interactions (2CDU)	α-Gurjunene (−7.2 kcal/mol), Alloaromadendrene (−7.1 kcal/mol), α-Muurolene (−7.1 kcal/mol), 1,5-Cadinadiene (−7.1 kcal/mol), and (−)-β-Bourbonene (−7.0 kcal/mol). Reference ligand (Oxacillin): −8.6 kcal/mol.
Best antifungal docking interactions (4DKI)	trans-α-Bisabolene (−8.0 kcal/mol), (−)-β-Bourbonene (−7.8 kcal/mol), α-Cubebene (−7.5 kcal/mol), β-Copaene (−7.4 kcal/mol), and Ylangenal (−7.4 kcal/mol). Reference ligand (Fluconazole): −6.9 kcal/mol.
Best antibacterial docking interactions (5TZ1)	1,5-Cadinadiene (−7.1 kcal/mol), (−)-β-Bourbonene (−7.0 kcal/mol), Isogermacrene D (−6.9 kcal/mol), Alloaromadendrene (−6.8 kcal/mol), and trans-α-Bisabolene (−6.8 kcal/mol). Reference ligand (Vitamin C): −6.2 kcal/mol.
Overall conclusion	The carvone-rich essential oil of L. alba exhibited antioxidant, antifungal, and antibacterial activities supported by molecular docking analyses, providing scientific support for its traditional medicinal use.

## Data Availability

The data presented in this study are available within the article.

## Abbreviations

The following abbreviations are used in this manuscript:

EOLA	essential oil of *Lippia alba*
DPPH	2,2-diphenyl-1-picrylhydrazyl
ABTS	2,2′-azino-bis-3-ethylbenzothiazoline-6-sulfonic acid
GC–MS	gas chromatography–mass spectrometry;
TE	Trolox equivalent
ADMET	absorption, distribution, metabolism, excretion, and toxicity
PBP2a	penicillin-binding protein 2a
CYP51	sterol 14-α-demethylase
MRSA	methicillin-resistant *Staphylococcus aureus*
